# Efficacy and safety of low-dose rituximab in MuSK antibody-positive myasthenia gravis: a single-center retrospective study

**DOI:** 10.3389/fneur.2026.1929854

**Published:** 2026-08-24

**Authors:** Li Yang, Zhenyu Wu, Liqing Hu, Yufei Deng, Haocheng Luo, Xuxiang Zhang, Xiaojun Yang, Jiang Qilong

**Affiliations:** 1 Guangzhou University of Chinese Medicine, Guangzhou, China; 2 Guangzhou University of Traditional Chinese Medicine First Affiliated Hospital, Guangzhou, China

**Keywords:** B-cell depletion, CD19+ B cells, low-dose therapy, MuSK antibody, myasthenia gravis, rituximab

## Abstract

**Background:**

Rituximab (RTX) is increasingly used for refractory myasthenia gravis (MG), particularly muscle-specific kinase antibody-positive MG (MuSK-MG). However, the optimal dosing regimen remains to be standardized, and conventional high-dose protocols may increase treatment burden, cumulative exposure, and infection-related concerns. This study evaluated the clinical efficacy, safety, and immunological effects of a low-dose RTX regimen in patients with MuSK-MG.

**Methods:**

We retrospectively analyzed 35 patients with MuSK-MG who received low-dose RTX at the First Affiliated Hospital of Guangzhou University of Chinese Medicine between January 1, 2017 and August 1, 2025. RTX was administered as 100 mg on day 1 and 500 mg on day 2, and the regimen was repeated every 6 months. All patients received at least two treatment cycles. Clinical outcomes were assessed using Myasthenia Gravis Activities of Daily Living (MG-ADL) scores at baseline and at 3, 6, and 12 months after treatment. Secondary outcomes included Myasthenia Gravis Foundation of America post-intervention status (MGFA-PIS), adverse events, and peripheral CD19+ B-cell percentages in 24 patients.

**Results:**

The mean baseline MG-ADL score was 9.5 ± 5.6 and decreased to 2.1 ± 2.3 at 3 months, 1.0 ± 1.8 at 6 months, and 0.5 ± 0.9 at 12 months (all *p* < 0.001 vs. baseline). At 3, 6, and 12 months, 21 (60.0%), 25 (71.4%), and 28 (80.0%) patients achieved minimal manifestation status (MMS) or better was 6 months, respectively. Kaplan–Meier analysis showed a median time to MMS or better of 6 months. Both the bulbar involvement (BI) and non-bulbar involvement (non-BI) subgroups showed significant clinical improvement, although the trajectories of MG-ADL improvement differed between subgroups. In the 24 patients with available immunological data, the median CD19+ B-cell percentage decreased from 12.6% before treatment to 0.30% at 6 months (*p* < 0.001). Adverse events were mild, and no serious infusion reactions, severe infections, or hepatic or renal dysfunction were observed.

**Conclusion:**

Low-dose RTX was associated with rapid and sustained clinical improvement and effective peripheral B-cell depletion in patients with MuSK-MG, with acceptable tolerability. These findings support low-dose RTX as a potential therapeutic option for MuSK-MG, although prospective multicenter studies with standardized dosing and longer follow-up are needed.

## Introduction

Myasthenia gravis (MG) is an autoimmune disorder of neuromuscular transmission caused by autoantibodies targeting functional proteins at the postsynaptic membrane, most commonly the acetylcholine receptor (AChR) and muscle-specific kinase (MuSK) (1). MuSK antibody-positive MG accounts for approximately 5–8% of all MG cases and differs from AChR-positive MG in clinical phenotype, pathophysiology, and treatment response (2). Patients with MuSK-MG often present with prominent bulbar, neck, respiratory, and proximal limb weakness and may be at increased risk of myasthenic crisis (3). In addition, responses to acetylcholinesterase inhibitors and thymectomy are often limited, and these therapies may be poorly tolerated in some patients (3, 4).

Rituximab is a monoclonal antibody that targets CD20-positive B cells. By depleting mature B cells and modulating B-cell-mediated immune activity, RTX may reduce the production of pathogenic autoantibodies and modify disease activity in MuSK-MG (5, 6). Previous clinical studies and systematic reviews have suggested that RTX can induce clinical improvement or remission in a high proportion of patients with MuSK-MG and may reduce the need for glucocorticoids or other immunosuppressive therapies (7–10).

Despite these findings, the optimal RTX regimen for MuSK-MG remains uncertain. Many commonly used RTX regimens were adapted from lymphoma protocols, and they may increase treatment cost, cumulative exposure, and safety concerns. Low-dose RTX regimens have been explored in MG, but existing evidence is limited by small sample sizes, heterogeneous dosing protocols, relatively short follow-up periods, and insufficient subgroup analyses (11–13). Furthermore, whether clinical phenotypes such as bulbar involvement (BI) influence the response to low-dose RTX remains unclear.

Therefore, this single-center retrospective study evaluated the efficacy and safety of a standardized low-dose RTX regimen in patients with MuSK-MG. We also assessed peripheral CD19+ B-cell depletion and conducted an exploratory subgroup analysis according to the presence or absence of BI.

## Materials and methods

### Study design and patients

This study was designed as a single-center retrospective observational study. We reviewed the clinical data of 35 patients with MuSK-MG who received low-dose RTX at the First Affiliated Hospital of Guangzhou University of Chinese Medicine from January 1, 2017 to August 1, 2025.

The inclusion criteria were as follows: (1) age 18 years or older; (2) clinical symptoms and electrophysiological findings consistent with MG; (3) positive MuSK antibody and negative AChR antibody; (4) receipt of at least two cycles of RTX, and (5) no concomitant autoimmune diseases. The study was approved by the Research Ethics Committee of the First Affiliated Hospital of Guangzhou University of Chinese Medicine and was conducted in accordance with the Declaration of Helsinki.

Additional exclusion criteria were applied to minimize potential misclassification of BI. Patients were excluded if available clinical records indicated alternative neurological, structural, or metabolic causes of dysphagia or dysarthria, including Parkinson’s disease, motor neuron disease, cranial neuropathies, polyneuropathies, primary myopathies, head and neck malignancies, cervical structural lesions, vitamin B12 deficiency, diabetes-related neuropathy, or other metabolic disorders.

### Clinical data collection and outcome assessment

Demographic and clinical data were collected, including sex, age, age at onset, disease duration, clinical manifestations, baseline MGFA clinical classification, previous treatments, number of myasthenic crises, and baseline MG-ADL score. The date of the first RTX infusion was defined as baseline. Follow-up assessments were performed at 3, 6, and 12 months after RTX initiation.

The primary outcome was the change in MG-ADL score from baseline to each follow-up time point. Secondary outcomes included MGFA-PIS, time to MMS or better, adverse events, and peripheral CD19+ B-cell percentage. In this study, MMS or better was operationally defined as a final MG-ADL score of 0 or 1. Clinical improvement was defined as a decrease of more than 3 points in MG-ADL score compared with baseline. A decrease of 3 points or less was considered unchanged, and an increase in MG-ADL score was defined as clinical worsening. Adverse events were collected from medical records and graded according to the Common Terminology Criteria for Adverse Events when sufficient information was available.

Patients were classified into two exploratory subgroups according to the presence of BI: the BI group and the non-BI group. The terms BI and non-BI are used consistently throughout the manuscript.

BI was defined as clinically documented dysphagia, dysarthria, chewing weakness, or other bulbar symptoms judged by the treating neurologist to be attributable to MG.

### Rituximab treatment regimen

**Figure 1:**
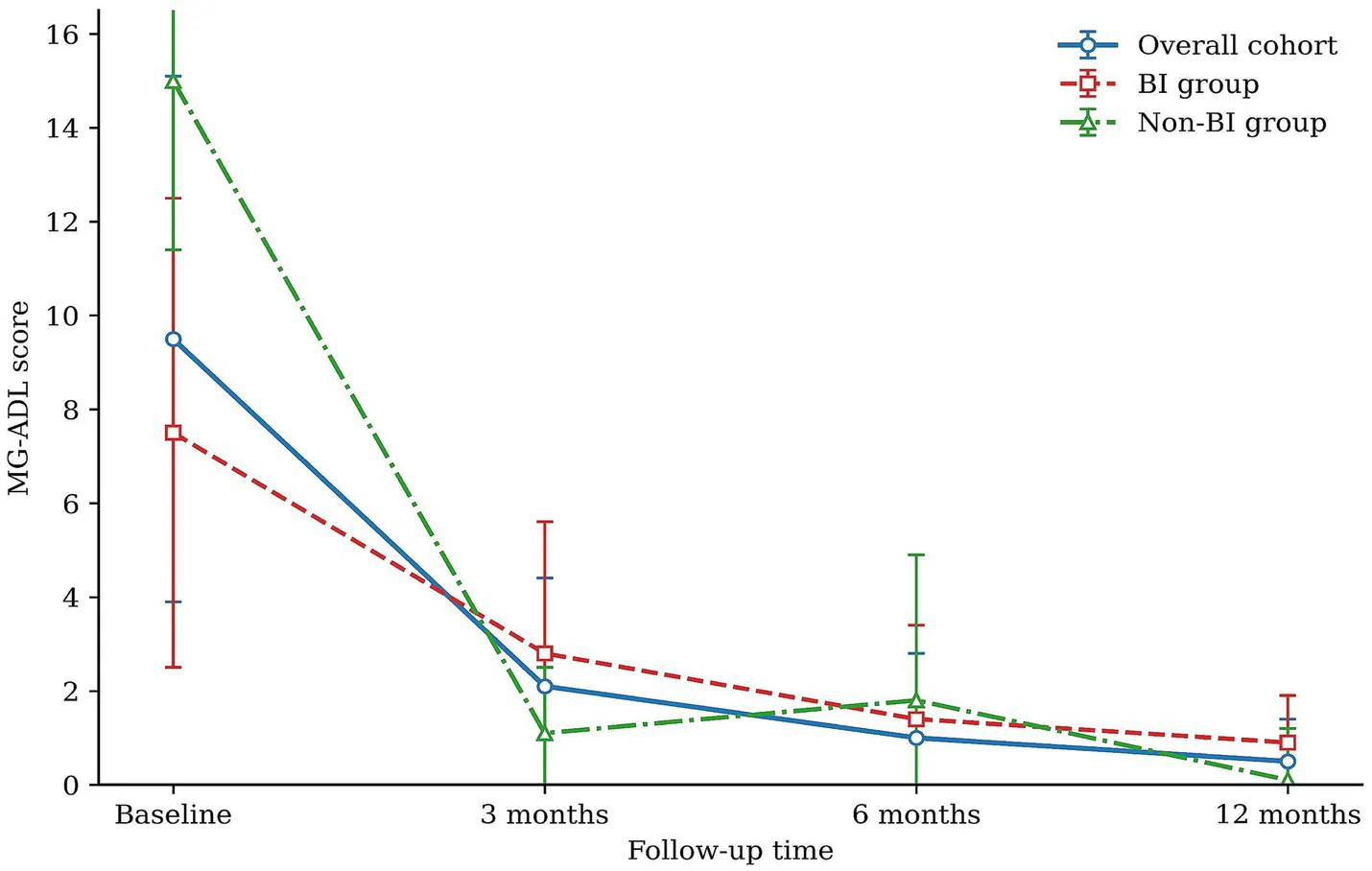
Standard line plot of MG-ADL scores from baseline to 3, 6, and 12 months after low-dose rituximab treatment in the overall cohort, BI group, and non-BI group. Values are presented as mean ± SD.

**Figure 2:**
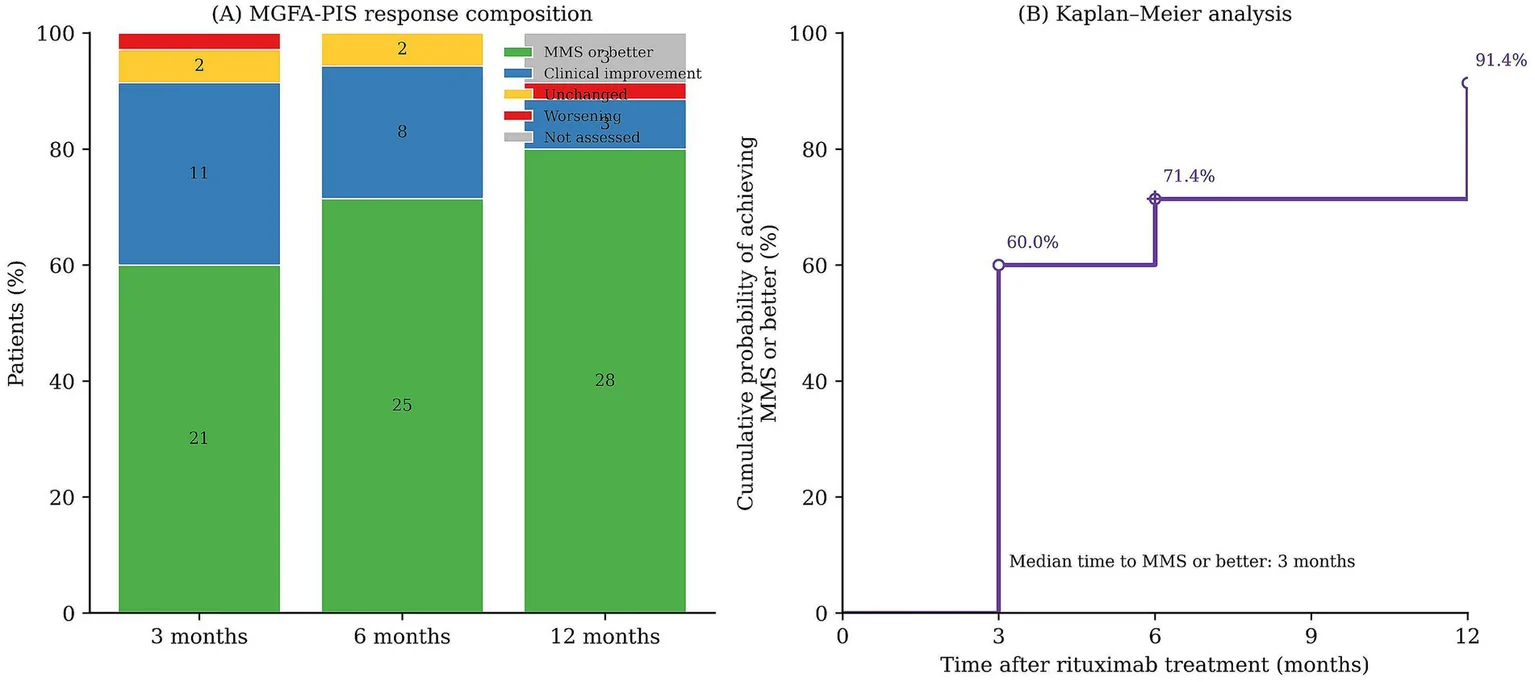
Clinical response after low-dose rituximab treatment. **(A)** Stacked bar chart showing the distribution of MGFA-PIS categories at 3, 6, and 12 months. **(B)** Kaplan–Meier curve showing the cumulative probability of achieving minimal manifestation status (MMS) or better was 6 months.

**Figure 3:**
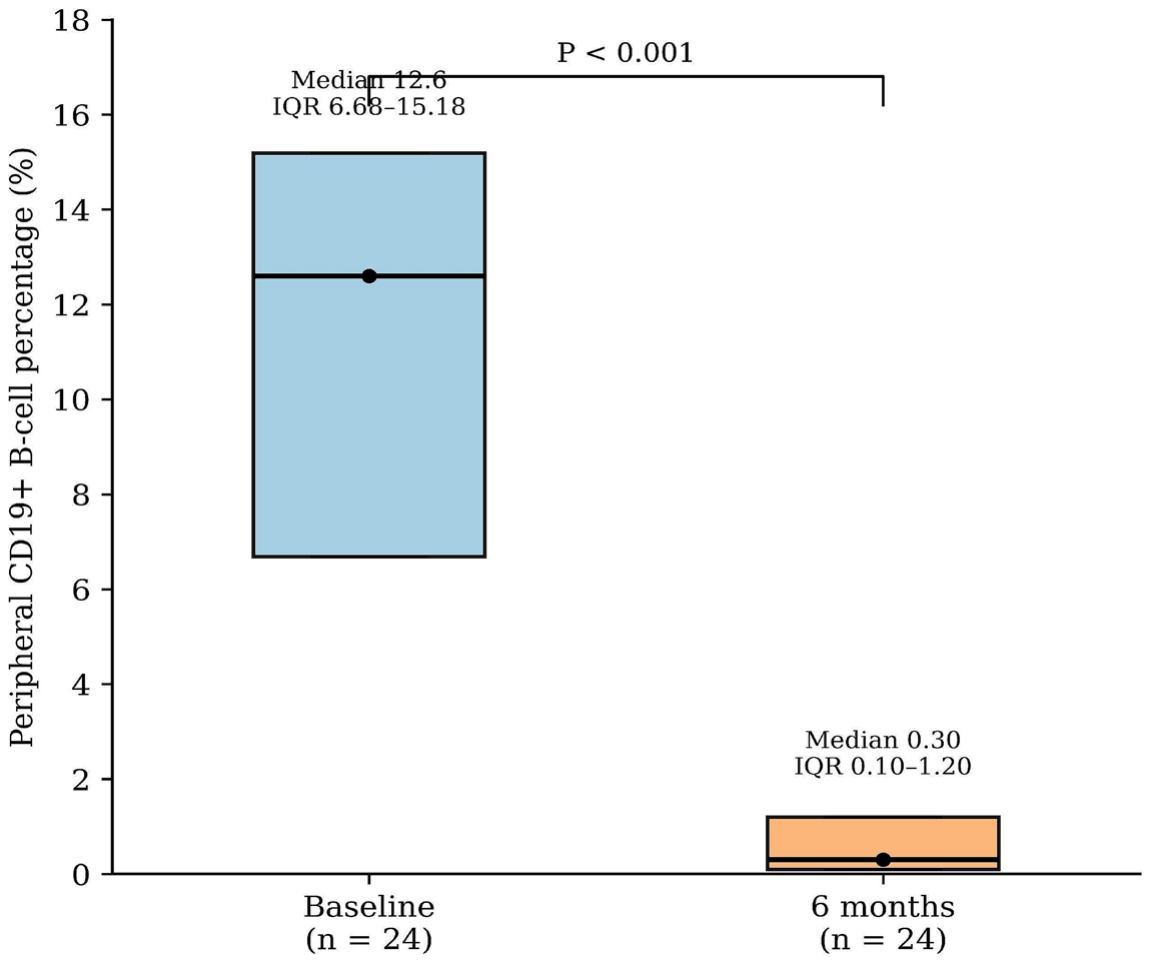
Standard box plot of peripheral CD19+ B-cell percentages at baseline and 6 months after low-dose rituximab treatment in the 24 patients with paired data. Boxes indicate interquartile range, and central lines indicate the median.

Before RTX treatment, patients had received acetylcholinesterase inhibitors, glucocorticoids, nonsteroidal immunosuppressants, intravenous immunoglobulin, or plasma exchange according to clinical need. When RTX was initiated, concomitant treatments were continued or tapered according to the treating physician’s judgment and clinical response. RTX was administered on two consecutive days: 100 mg on day 1 and 500 mg on day 2. The same regimen was repeated every 6 months. All included patients completed at least two treatment cycles. Because tapering schedules were not standardized in this retrospective cohort, concomitant therapies were considered potential confounders when interpreting clinical and immunological responses (Figures 1–3).

### Peripheral B-cell measurement

The percentage of peripheral blood CD19+ B cells was measured by flow cytometry at baseline and 6 months after RTX treatment in 24 patients with available paired data.

### Statistical analysis

Statistical analyses were performed using SPSS version 27.0. Continuous variables were tested for normality before analysis. Normally distributed variables are presented as mean ± standard deviation (SD), and non-normally distributed variables are presented as median and interquartile range (IQR). Categorical variables are presented as counts and percentages. Repeated-measures analysis of variance was used to compare MG-ADL scores across time points, with Bonferroni correction for *post hoc* comparisons when appropriate. Kaplan–Meier analysis was used to estimate the cumulative probability and median time to MMS or better. Because CD19+ B-cell percentages were not normally distributed, paired comparisons before and after RTX treatment were performed using the Wilcoxon signed-rank test. A two-sided *p* value <0.05 was considered statistically significant. Subgroup analyses were considered exploratory because of the limited sample size. Additional exploratory considerations included baseline MGFA clinical severity, concomitant glucocorticoid or nonsteroidal immunosuppressant use, and the relationship between CD19+ B-cell depletion and clinical response. Class-by-class MGFA comparisons and multivariable modeling were not performed because of the limited and uneven subgroup sizes.

## Results

### Baseline clinical characteristics

Thirty-five patients with MuSK-MG were included; 30 were women (85.7%) and 5 were men (14.3%). The mean age was 47.9 ± 13.4 years, the mean age at onset was 41.25 ± 12.67 years, and the mean disease duration was 6.5 ± 5.7 years. The mean number of myasthenic crises was 1.1 ± 1.2.

Twenty-four patients (68.5%) were classified into the BI group, and 11 patients (31.5%) were classified into the non-BI group. Baseline MGFA clinical classifications were class I in 1 patient (2.8%), class II in 24 patients (68.6%), class III in 8 patients (22.8%), and class IV in 2 patients (5.7%); no patients were classified as class V. Before RTX treatment, 32 patients (91.4%) had received acetylcholinesterase inhibitors, 30 (85.7%) had received glucocorticoids, 22 (62.8%) had received nonsteroidal immunosuppressants, 9 (25.7%) had received plasma exchange, and 4 (11.4%) had received intravenous immunoglobulin. The overall baseline MG-ADL score was 9.5 ± 5.6. Baseline characteristics are summarized in Table 1.

**Table 1:** Baseline clinical characteristics of the 35 patients with MuSK-MG.

Clinical variable	Value
Age, years	47.9 ± 13.4
Female sex, *n* (%)	30 (85.7%)
Male sex, *n* (%)	5 (14.3%)
Age at onset, years	41.25 ± 12.67
Disease duration, years	6.5 ± 5.7
Number of myasthenic crises	1.1 ± 1.2
Bulbar involvement group, *n* (%)	24 (68.5%)
Non-bulbar involvement group, *n* (%)	11 (31.5%)
MGFA class I, *n* (%)	1 (2.8%)
MGFA class II, *n* (%)	24 (68.6%)
MGFA class III, *n* (%)	8 (22.8%)
MGFA class IV, *n* (%)	2 (5.7%)
MGFA class V, *n* (%)	0 (0%)
Previous cholinesterase inhibitor use, *n* (%)	32 (91.4%)
Previous glucocorticoid use, *n* (%)	30 (85.7%)
Previous non-steroidal immunosuppressant use, *n* (%)	22 (62.8%)
Previous plasma exchange, *n* (%)	9 (25.7%)
Previous intravenous immunoglobulin, *n* (%)	4 (11.4%)
Baseline MG-ADL score	9.5 ± 5.6

### Changes in MG-ADL scores after treatment

Repeated-measures analysis of variance showed a significant time effect in the overall cohort (*f* = 93.743, *p* < 0.001). The MG-ADL score decreased from 9.5 ± 5.6 at baseline to 2.1 ± 2.3 at 3 months, 1.0 ± 1.8 at 6 months, and 0.5 ± 0.9 at 12 months after RTX treatment (all *p* < 0.001 vs. baseline) (Table 2).

**Table 2:** MG-ADL score changes before and after low-dose rituximab treatment.

Time point	Overall cohort	BI group	Non-BI group	Between-group *p* value
Baseline	9.5 ± 5.6	7.5 ± 5.0	15.0 ± 3.6	<0.001
3 months	2.1 ± 2.3	2.8 ± 2.8	1.1 ± 1.4	0.081
6 months	1.0 ± 1.8	1.4 ± 2.0	1.8 ± 3.1	0.251
12 months	0.5 ± 0.9	0.9 ± 1.0	0.1 ± 1.1	0.378
Within-group time effect	*f* = 93.743, *p* < 0.001	*f* = 13.33, *p* < 0.001	*f* = 33.33, *p* < 0.001	–
Time × group interaction	–	–	–	*f* = 15.014, *p* < 0.001

BI, bulbar involvement; non-BI, non-bulbar involvement. Values are presented as mean ± SD unless otherwise indicated. The subgroup analysis was exploratory.

In exploratory subgroup analysis, both the BI and non-BI groups showed significant reductions in MG-ADL scores over time. The interaction between time and subgroup was significant (*f* = 15.014, *p* < 0.001), suggesting different trajectories of improvement. Because baseline MG-ADL scores differed between subgroups, these findings should be interpreted cautiously.

### Clinical response according to MGFA-PIS

At 3 months after RTX treatment, 21 patients (60.0%) achieved MMS or better, 11 (31.4%) showed clinical improvement, 2 (5.7%) were unchanged, and 1 (2.9%) showed clinical worsening. At 6 months, 25 patients (71.4%) achieved MMS or better, 8 (22.9%) showed clinical improvement, and 2 (5.7%) were unchanged. At 12 months, 28 patients (80.0%) achieved MMS or better, 3 (8.5%) showed clinical improvement, and 1 (2.8%) showed clinical worsening.

Kaplan–Meier analysis showed that the median time to MMS or better was 6 months. The cumulative probability of achieving MMS or better was 54.3% (19/35) within 5 months and exceeded 90.0% by 12 months. In subgroup analysis, the proportions of patients achieving MMS or better in the BI group were 66.7, 75.0, and 91.6% at 3, 6, and 12 months, respectively; the corresponding proportions in the non-BI group were 45.5, 63.6, and 72.7%. Differences between subgroups were not statistically significant at any time point (all *p* > 0.05).

Exploratory considerations regarding baseline severity and concomitant immunotherapy.

Baseline MGFA severity was not analyzed by individual class because of the small and uneven number of patients in each category, particularly class I and class IV, and the absence of class V cases. In addition, most patients had received glucocorticoids and nearly two-thirds had received nonsteroidal immunosuppressants before RTX initiation. These factors limited reliable subgroup comparisons and were therefore interpreted as potential confounders rather than independent predictors in the present analysis.

### Peripheral CD19+ B-cell depletion

Paired data on peripheral CD19+ B-cell percentages were available for 24 patients. The median CD19+ B-cell percentage decreased from 12.6% (IQR, 6.68–15.18%) at baseline to 0.30% (IQR, 0.10–1.20%) at 6 months after RTX treatment. The difference was statistically significant using the Wilcoxon signed-rank test (*p* < 0.001), indicating effective peripheral B-cell depletion after the low-dose RTX regimen.

Because CD19+ B-cell percentages were measured only at baseline and 6 months and paired data were available for 24 patients, the present study could not robustly evaluate B-cell kinetics, determine whether the magnitude of depletion predicted achievement of MMS or better, or compare longitudinal depletion patterns between the BI and non-BI groups.

### Safety

Overall, adverse events were mild and manageable and were reported in 5 patients (14.3%). Reported events included limb numbness (*n* = 3, 8.6%), chest tightness (*n* = 1, 2.9%), morning stiffness (*n* = 1, 2.9%), and alopecia (*n* = 1, 2.9%). At the final follow-up, isolated events included morning stiffness (*n* = 1, 2.9%), hyperglycemia (*n* = 1, 2.9%), infection (*n* = 1, 2.9%), and pruritus (*n* = 1, 2.9%). No serious infusion reactions, severe infections, hepatic or renal dysfunction, or allergic reactions were documented. The discrepancy between the number of affected patients and the number of event types reflects that one patient may have experienced more than one mild event.

## Discussion

This single-center retrospective study suggests that a standardized low-dose RTX regimen was associated with rapid and sustained clinical improvement in patients with MuSK-MG. The MG-ADL score decreased significantly by 3 months and remained low at 6 and 12 months. In addition, most patients achieved MMS or better during follow-up, and paired immunological data showed marked depletion of peripheral CD19+ B cells at 6 months. The treatment was generally well tolerated, with no serious adverse events documented in the available records.

MuSK-MG is an IgG4-mediated autoimmune disease in which B cells and antibody-producing plasmablast-lineage cells are thought to play important roles (14–16). RTX exerts its therapeutic effect mainly by depleting CD20-positive B cells and modulating B-cell-dependent immune responses (17, 18). However, the relationship between peripheral B-cell depletion and clinical efficacy is not fully established. Previous studies have reported that some patients improve clinically without complete peripheral B-cell depletion, whereas others may relapse despite sustained depletion (19). In the present study, the significant decrease in CD19+ B-cell percentage supports the biological activity of the low-dose regimen, but we could not determine whether the magnitude or duration of depletion predicted clinical response. Future studies should include MuSK antibody titers, memory B-cell subsets, plasmablasts, and longitudinal immune-reconstitution profiles. Specifically, we could not determine whether the magnitude of CD19+ B-cell depletion correlated with MG-ADL improvement, whether the BI and non-BI groups exhibited different B-cell depletion patterns, or whether early B-cell kinetics predicted achievement of MMS or better.

Several studies have supported the efficacy of RTX in MuSK-MG, and international guidance increasingly recognizes RTX as an important option in patients with inadequate responses to conventional immunotherapy (20–23). Nevertheless, the most appropriate RTX dose remains uncertain. Traditional regimens are often adapted from lymphoma protocols, whereas lower-dose regimens may reduce treatment burden and cost while maintaining clinical efficacy. Our regimen consisted of 600 mg per cycle given over two consecutive days and repeated every 6 months. The high proportion of patients achieving MMS or better is consistent with prior reports suggesting favorable responses to RTX in MuSK-MG (24–26). However, because this study did not include a high-dose RTX control group, the results should not be interpreted as evidence that low-dose RTX is superior to high-dose RTX. Rather, the findings provide real-world evidence that this low-dose regimen may be a feasible and well-tolerated option.

The exploratory subgroup analysis according to BI is clinically relevant because patients with MuSK-MG frequently present with bulbar and respiratory symptoms (27). Both patients with and without BI improved after treatment, but the trajectories of MG-ADL improvement differed between groups. This finding may reflect underlying clinical heterogeneity, differences in baseline disease severity, or the limited sample size. Because baseline MG-ADL scores differed significantly between the two subgroups, the subgroup results should be interpreted as hypothesis-generating rather than confirmatory. Although available records were reviewed for alternative causes of dysphagia or dysarthria, the retrospective design precluded a uniform prospective screening protocol for all patients; therefore, residual misclassification of BI cannot be completely excluded. Baseline MGFA clinical severity may also influence treatment response, but class-specific comparisons were underpowered because individual MGFA classes were unevenly represented. Larger prospective studies are needed to determine whether BI, baseline MGFA severity, or other clinical phenotypes predict response, relapse risk, or the need for repeated RTX dosing.

Safety is an important consideration when selecting an RTX regimen for MuSK-MG. Long-term glucocorticoid exposure may cause osteoporosis, metabolic complications, and infection risk, while conventional immunosuppressants may be limited by delayed onset, adverse effects, or relapse during dose reduction (28, 29). In this study, adverse events were mild, and no serious infusion reactions or severe infections were documented. However, the retrospective design and limited follow-up restrict our ability to assess uncommon or delayed adverse events, including hypogammaglobulinemia, impaired vaccine response, opportunistic infections, and long-term immune reconstitution. Systematic monitoring of immunoglobulin levels and infection outcomes should be incorporated into future studies. Some events, such as chest tightness and alopecia, were conservatively recorded as adverse events based on the available medical records; however, their causal relationship with RTX could not be firmly established. These manifestations may overlap with non-motor or systemic features of MG and should therefore be interpreted cautiously (30).

This study has several limitations. First, the retrospective single-center design and small sample size may introduce selection bias and limit generalizability. Second, the absence of a control group means that the observed improvement cannot be attributed solely to RTX, especially because some patients continued glucocorticoids or other immunotherapies; detailed dose changes and tapering schedules were not uniformly available, and concomitant therapies may have influenced both clinical outcomes and immune markers. Third, paired CD19+ B-cell data were available only for 24 patients, CD19+ B-cell percentages were measured at only two time points, and MuSK antibody titers and detailed B-cell subsets were not assessed. Therefore, we could not determine whether CD19+ B-cell depletion kinetics predicted clinical response or MMS achievement. Fourth, the subgroup analysis was exploratory and underpowered, particularly because baseline MG-ADL scores differed between the BI and non-BI groups and MGFA clinical classes were unevenly distributed. Fifth, although alternative causes of BI were reviewed using available medical records, the retrospective design limited systematic exclusion of all neurological, structural, or metabolic causes of dysphagia and dysarthria. Sixth, the follow-up period was limited to 12 months for the main outcomes, which is insufficient to fully evaluate relapse, optimal retreatment intervals, cumulative safety, and long-term remission. Prospective multicenter studies with standardized tapering protocols, immune monitoring, and longer follow-up are needed to confirm these findings.

## Conclusion

Low-dose RTX was associated with rapid and sustained improvement in MG-ADL scores, a high proportion of patients achieving MMS or better, and significant peripheral CD19+ B-cell depletion in patients with MuSK-MG. The regimen appeared to be well tolerated in this retrospective cohort. These findings support low-dose RTX as a potential therapeutic option for MuSK-MG, particularly in patients with inadequate responses to conventional therapy. Further multicenter prospective studies are required to confirm efficacy and safety, define optimal dosing and retreatment strategies, and identify predictive biomarkers such as MuSK antibody titers and B-cell subsets.

## Data Availability

The original contributions presented in the study are included in the article/supplementary material, further inquiries can be directed to the corresponding author.
